# Safety and Efficacy of a Phage, kpssk3, in an *in vivo* Model of Carbapenem-Resistant Hypermucoviscous *Klebsiella pneumoniae* Bacteremia

**DOI:** 10.3389/fmicb.2021.613356

**Published:** 2021-05-20

**Authors:** Yunlong Shi, Yuan Peng, Yixin Zhang, Yu Chen, Cheng Zhang, Xiaoqiang Luo, Yajie Chen, Zhiqiang Yuan, Jing Chen, Yali Gong

**Affiliations:** ^1^State Key Laboratory of Trauma, Burns and Combined Injury, Institute of Burn Research, Southwest Hospital, Third Military Medical University (Army Medical University), Chongqing, China; ^2^Department of Plastic and Reconstructive Surgery, Shanghai Ninth People’s Hospital, Shanghai Jiao Tong University School of Medicine, Shanghai, China

**Keywords:** phage, *Klebsiella pneumoniae*, gut microbiota, hypermucoviscous, carbapenem-resistant

## Abstract

Antimicrobial resistance (AMR) is one of the most significant threats to global public health. As antibiotic failure is increasing, phages are gradually becoming important agents in the post-antibiotic era. In this study, the therapeutic effects and safety of kpssk3, a previously isolated phage infecting carbapenem-resistant hypermucoviscous *Klebsiella pneumoniae* (CR-HMKP), were evaluated in a mouse model of systemic CR-HMKP infection. The therapeutic efficacy experiment showed that intraperitoneal injection with a single dose of phage kpssk3 (1 × 10^7^ PFU/mouse) 3 h post infection protected 100% of BALB/c mice against bacteremia induced by intraperitoneal challenge with a 2 × LD_100_ dose of NY03, a CR-HMKP clinical isolate. In addition, mice were treated with antibiotics from three classes (polymyxin B, tigecycline, and ceftazidime/avibactam plus aztreonam), and the 7 days survival rates of the treated mice were 20, 20, and 90%, respectively. The safety test consisted of 2 parts: determining the cytotoxicity of kpssk3 and evaluating the short- and long-term impacts of phage therapy on the mouse gut microbiota. Phage kpssk3 was shown to not be cytotoxic to mammalian cells *in vitro* or *in vivo*. Fecal samples were collected from the phage-treated mice at 3 time points before (0 day) and after (3 and 10 days) phage therapy to study the change in the gut microbiome via high-throughput 16S rDNA sequence analysis, which revealed no notable alterations in the gut microbiota except for decreases in the Chao1 and ACE indexes.

## Introduction

Carbapenem-resistant *Klebsiella pneumoniae* (CRKP) has recently become one of the most important global public health challenges, causing treatment failures, high mortality rates, exorbitant healthcare expenses and prolonged hospital stays (Suay-Garcia and Perez-Gracia, 2019). In 2017, the World Health Organization (WHO) listed CRKP as a high-priority critical pathogen due to the limited treatment options and urgent need for new drugs (Venter, 2019). Some traditional antibiotics, e.g., polymyxin and tigecycline, remain drugs of last resort against CRKP, but the optimization of dosage regimens, their high toxicity, low efficacy and increasing resistance are issues remaining to be addressed (Porreca et al., 2018). Ceftazidime/avibactam (CAZ/AVI) is an effective antimicrobial combination that was approved for clinical use in 2015 and provides a new alternative strategy to treat CRKP; however, CAZ/AVI resistance was reported even before this treatment was commercially available in China (Zhang et al., 2020). In addition, in 1986, a report from Taiwan first described a new *Klebsiella pneumoniae* (*K. pneumoniae*) strain with a high-virulence and hypermucoviscosity phenotype—i.e., hypervirulent *K. pneumoniae* (hvKP)—that could cause serious invasive infections, such as endophthalmitis, pyogenic liver abscess (PLA) and meningitis, in relatively young and healthy individuals (Gu et al., 2018). Even more concerningly, an increasing number of *K. pneumoniae* clinical isolates with a combination of carbapenem resistance and hypervirulence are being reported worldwide, which might cause a severe public health crisis (Chen and Kreiswirth, 2018).

Bacteriophages (phages) are viruses that can kill specific bacteria (Guo et al., 2020). Approximately one century ago, phages were discovered and used as antibacterial agents to combat infections. During World War II, phage therapy (PT) saved the lives of many soldiers (Myelnikov, 2018). Compared to antibiotic therapy, PT has many advantages, such as strict specificity toward target pathogens, the ability of phages to self-replicate, the ability to eradicate bacterial biofilms, low toxicity, and relatively few side effects, and is thus an alternative treatment modality for infectious diseases in the postantibiotic era. However, before PT can be introduced as a therapeutic approach for bacterial infection in humans, its efficacy and safety must be evaluated in an animal model. In previous work, using a CR-HMKP strain (NY03) isolated from blood samples of a patient with severe burns as the host, we isolated a T7-like lytic phage from hospital sewage (Shi et al., 2020). Here, based on this previous work, we evaluated the therapeutic efficacy and safety of this phage in a mouse model.

## Materials and Methods

### Ethics Statement

All animal experiments in this study were approved by the Ethics Committee of Army Medical University (AMU), Chongqing, China. The BALB/c female mice (18–22 g, 6–8 weeks old) used in the study were purchased from the Experimental Animal Center of AMU and kept in a room maintained at 24 ± 3°C with free access to a standard rodent diet and sterile drinking water on a 12 h light/dark cycle. All mice were euthanized at the end of the experiments.

### Bacterial Strain and Phage

Both the phage kpssk3 and the CRKP clinical isolate NY03 used in this study were previously characterized and analyzed (Shi et al., 2020). NY03 was screened by PCR for the presence of resistance genes, as previously described (Vergara et al., 2020). NY03 was grown in Luria–Bertani broth at 37°C. An endotoxin affinity column (ToxinEraser Endotoxin Removal Kit, Genscript, China) was used to remove endotoxin from the phage preparation, which reduced the endotoxin-to-phage ratio to less than 1 EU/10^9^ PFU (Hietala et al., 2019).

### Hospital Course of the Patient Infected With NY03

In 2017, a 15-years-old male patient with a severe electrical burn injury (total burn surface area = 75%) was hospitalized in a teaching hospital (Chongqing, China) for 50 days, during which he developed sepsis caused by CRKP (NY03). Specifically, on day 30 of hospitalization, culture of central blood revealed growth of a CRKP strain that was resistant to 14 antibiotics, including imipenem (IPM, MIC = 16 μg/ml), ertapenem (ETP, MIC = 8 μg/ml) and aztreonam (ATM, MIC = 64 μg/ml), and susceptible to polymyxin B (PMB, MIC = 1 μg/ml) and tigecycline (TGC, MIC = 1 μg/ml). However, CAZ/AVI was not commercially available in China at that time. The patient was treated with TGC and PMB; however, the CRKP strain was not eradicated from the circulatory system during the treatment course, and the patient’s overall condition deteriorated progressively. Finally, on day 50, the patient died of multiple organ failure (MOF) caused by CRKP.

### Antimicrobial Susceptibility Testing and Phenotype Detection

The susceptibility of NY03 to CAZ/AVI (Pfizer, Inc., United States) was tested by the broth microdilution method (BMD) according to the Clinical and Laboratory Standards Institute guidelines (28th edition) (Wang et al., 2020). NY03 carried a gene, *bla*_*GIM*_, encoding an Ambler class B metallo-β-lactamase (MBL), which might confer resistance to CAZ/AVI. A checkerboard assay was performed as previously described to determine the synergistic antibacterial effects of the combination of CAZ/AVI with ATM (Solarbio Life Science, China) against NY03 (Ungphakorn et al., 2018). The combination interaction of the 2 antibiotics was evaluated by calculating and interpreting the fractional inhibitory concentration index (FICI) according to criteria described elsewhere (Ferrer-Espada et al., 2020). Additionally, a string test was conducted as described previously to assess the hypermucoviscosity phenotypes of NY03. The formation of a viscous string with a length of at least 5 mm when the NY03 bacterial colony was stretched with a loop on a blood agar plate was considered to indicate a positive string test (Khalil et al., 2019). The presence of virulence genes, including *rmpA*/*rmpA*2, *magA*, *iucA*, and *iroA*, was checked by PCR using previously described methods (Alsanie, 2020).

### Cytotoxicity of kpssk3

First, the cytotoxicity of different concentrations of kpssk3 (1 × 10^8^ PFU/ml and 1 × 10^9^ PFU/ml) to HeLa cells was evaluated with a Cell Counting Kit-8 (CCK-8, Sigma, Japan) in accordance with the instructions (Aleksandrzak et al., 2019). Triton X-100 (2%) was used as the positive control, and PBS buffer was used as the negative control. The absorbance (optical density, OD) at 450 nm (A_45__0n__*m*_) was measured in a microplate reader (Thermo Fisher Scientific, United States). For *in vivo* studies, 2 different doses of kpssk3 (10^8^ PFU/mouse and 10^9^ PFU/mouse) were injected into 10 mice (5 mice for each dose level) intraperitoneally (i.p.) twice daily for 5 days. During the experimental period, the health status of the mice was monitored based on the following symptoms: decreased physical activity, lethargy, hunched posture, unkempt fur, purulent periocular secretions, labored breathing, and death (Xia et al., 2016), and the body weight was recorded daily. Two randomly selected mice (1 for each dose level) were euthanized by cervical dislocation after 5 days, at which time the spleen, lungs, kidneys and liver were harvested for histopathological analysis.

### Distribution of kpssk3 in the Blood of Healthy Mice

Twenty-seven healthy mice were injected i.p. with 1 ml of 10^8^ PFU/ml kpssk3. At 6 min and at 1, 2, 3, 4, 5, 6, 7, and 8 h after injection, 3 mice were randomly selected and anesthetized by pentobarbital sodium (40 mg/kg, i.p.) (Ginosar et al., 2015). Cardiac puncture was then performed to collect approximately 1 ml of blood from the ventricle of each anesthetized mouse on which an open thoracotomy had been performed. The titer of kpssk3 in the blood was measured by the double-layer agar method as previously described (Fulgione et al., 2019). This experiment was repeated three times.

### PT in a CR-HMKP-Induced Bacteremia Model

**FIGURE 1:**
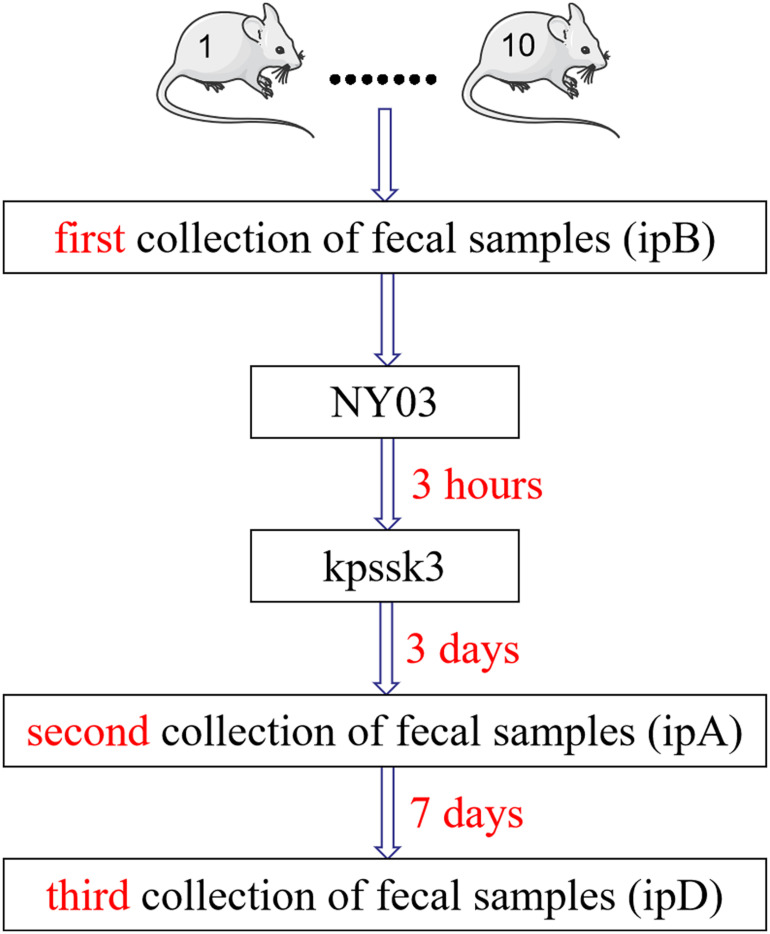
Diagrammatic sketch of the fecal sample collection process.

First, the absolute lethal dose (LD_100_) of strain NY03 in mice was determined as described elsewhere (Cheng et al., 2017). The model of CR-HMKP-induced bacteremia was established by intraperitoneal injection of a 2 × LD_100_ dose of early log-phase NY03, which was followed 3 hours later by treatment with different antibacterial agents. Sixty mice were averagely divided into 6 groups and treated as follows:

(Group 1) Mice were infected i.p. with 0.1 ml of NY03 (2 × LD_100_/mouse);
(Group 2) Mice were injected subcutaneously (s.c.) with 10 mg/kg PMB (Sangon, Shanghai, China) twice daily after bacterial challenge (Landersdorfer et al., 2018);
(Group 3) Mice were injected s.c. with 5 mg/kg TGC (Pfizer, Inc., United States) twice daily after bacterial challenge (Docobo-Perez et al., 2012);
(Group 4) Mice were treated with a single dose of kpssk3 (0.1 ml, 1 × 10^8^ PFU/ml, i.p.). Simultaneously, we also collected feces from the mice of this group to study the changes in the mice gut microbiome during the treatment period (Figure 1);
(Group 5) Mice were treated with a single dose of kpssk3 (0.1 ml, 1 × 10^7^ PFU/ml, i.p.);
(Group 6) Mice were treated with a combined regimen of CAZ/AVI (32 mg/kg every 8 h, s.c.) plus ATM (32 mg/kg every 8 h, s.c.) (Marshall et al., 2017);
(Group 7) Mice were treated with an equal amount of normal saline (0.1 ml, i.p.).

The survival rate in each group was recorded daily for 7 days. For PT, phage resistance is one major limitation, so the phage cross-streak assay was performed *in vitro* to screen phage-resistant mutant (Guo et al., 2017).

### PT’s Influence on the Mouse Gut Microbiota

We evaluated the therapeutic effects of kpssk3 in a CR-HMKP-induced bacteremia model in the previous step, and simultaneously collected fresh feces from the mice in group 4 three times in a sterile manner to study the alterations in the mouse gut microbiome after PT (Figure 1). The fecal samples (approximately 1 g) were harvested by gently massaging the abdomens of the mice and were immediately frozen at −80°C. The mice in group 4 were housed in 10 numbered cages (1–10). The first 10 fecal samples were collected before infection with NY03 and designated ipB (1–10). The second set of fecal samples was collected 3 days after phage treatment and designated ipA (1–10). The third set of fecal samples was collected after 7 more days, i.e., 10 days after kpssk3 treatment, and designated ipD (1–10). After 10 days, one surviving mouse was randomly selected from group 4 and euthanized for histopathological analysis; the remaining mice in group 4 were sacrificed to determine the presence of kpssk3 and NY03 in the blood collected using the cardiac puncture method and different organs (heart, liver, spleen, lungs, kidneys and brain). Blood and organs homogenized in 2 ml of normal saline were then streaked onto Columbia blood agar base plates containing 5% defibrinated sheep blood and incubated for 12 h at 37°C. The presence of kpssk3 was detected using the double-layer agar method.

### 16S rDNA Sequencing and Data Analysis

After microbial DNA was extracted from the 30 fecal samples using HiPure Soil DNA Kits (Magen, Guangzhou, China) according to the specifications, the V3 and V4 regions of 16S rDNA were amplified by PCR as previously described (Guo et al., 2017; Lv et al., 2020). The PCR products extracted from the 2% agarose gel were then purified, quantified, and sequenced (paired-end, 2 × 250 bp) on the Illumina MiSeq platform. Raw reads were filtered with FASTP^1^ to obtain clean reads, which were further merged as raw tags with software FLASH (version 1.2.11) (Magoc and Salzberg, 2011). Raw tags were filtered with QIIME 1.9.1 software to obtain clean tags (Caporaso et al., 2010), and chimeric tags were then removed with UCHIME (version 8.1) to obtain effective tags. The UPARSE pipeline was used to identify operational taxonomic units (OTUs) with a sequence similarity of ≥ 97% (Edgar, 2013), and Venn analysis was performed using R Project 3.4.1 to identify the unique and common OTUs. Alpha diversity and beta diversity analyses were then carried out. The alpha diversity reflects the species diversity in a single sample and is evaluated using the Chao1, ACE, Simpson, and Shannon indexes, which were calculated with QIIME (version 1.9.1). The Chao1 and ACE indices are used to estimate the species richness of each group, which is proportional to the richness of each community, while the Simpson and Shannon indices demonstrate the species diversity, higher values of which indicate lower and greater species diversity, respectively. Alpha index comparison (*p*-value) was performed with the Wilcoxon rank sum test (between 2 groups) or Kruskal-Wallis test (among 3 groups) in R Project. Beta diversity is an important component of biological diversity, measuring compositional change across temporal and spatial scales. Beta diversity analysis was used to compare the composition of the microbial community among the different samples (Feng et al., 2019). Principal coordinates analysis (PCoA) and Adonis (all called PERMANOVA) tests were performed via R Project. *P* < 0.05 was considered statistically significant.

## Results

### Antimicrobial Susceptibility Testing and Phenotype Detection

**FIGURE 2:**
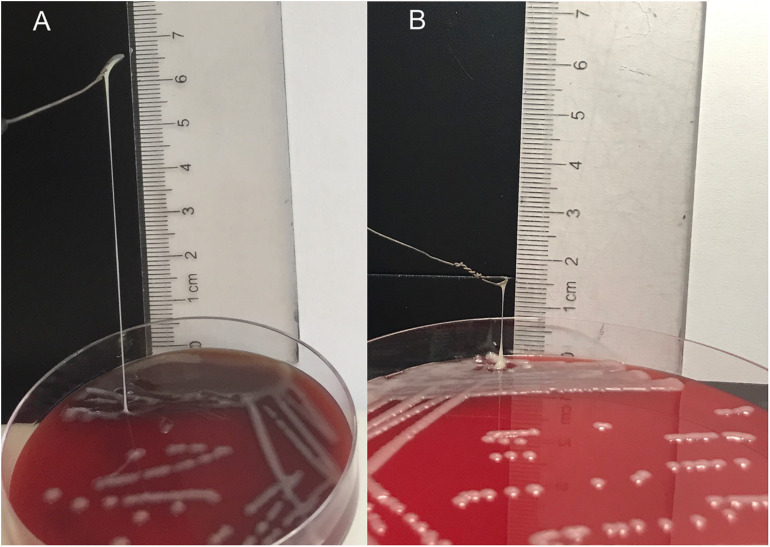
String test of NY03 **(A)** and NY03r **(B).**

Antibiotic resistance genes, including *bla*_*TEM*_, *bla*_*KPC*_, *bla*_*GIM*_, *bla*_*OXA*__–__48_, *bla*_*ACT*_, *bla*_*CTX*__–__*M*__–__9_, *bla*_*CTX*__–__*M*__–__10_, *bla*_*CTX*__–__*M*__–__14_, *bla*_*DHA*_, and *bla*_*SHV*_, were detected in NY03 by PCR. As expected, NY03 was resistant to CAZ/AVI with an MIC value of 32 mg/L. Table 1 showed the MICs of antibiotics alone and in combination. The combination of CAZ/AVI and ATM revealed a synergistic antibacterial effect against NY03 *in vitro* (FICI = 0.16). Regarding the identification of the hypermucoviscosity phenotype, NY03 generated a viscous filament of 6 cm in length when stretched with a loop (Figure 2), demonstrating that this NY03 colony with a positive string test possessed a hypermucoviscous phenotype and was thus considered carbapenem-resistant hypermucoviscous *K. pneumoniae* (CR-HMKP). However, NY03 was negative for the tested virulence genes.

**TABLE 1:** Results of MIC and AST of CAZ/AVI single dosing and combined with ATM against NY03.

MIC (mg/L) single dosing		MIC (mg/L) combined dosing		FIC value
ATM	CAZ-AVI	ATM	CAZ-AVI	
64	32	8	1	0.16

### Cytotoxicity of kpssk3

**FIGURE 3:**
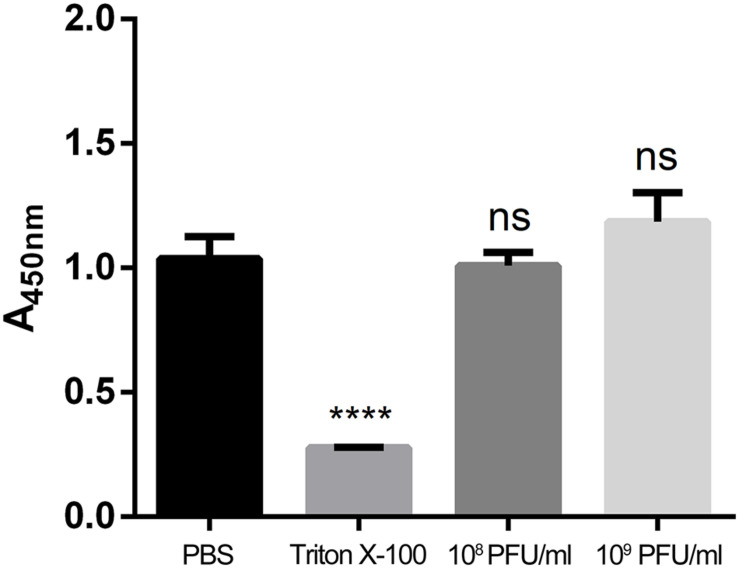
Cell viability evaluation by a CCK-8 assay (****P < 0.001).

**FIGURE 4:**
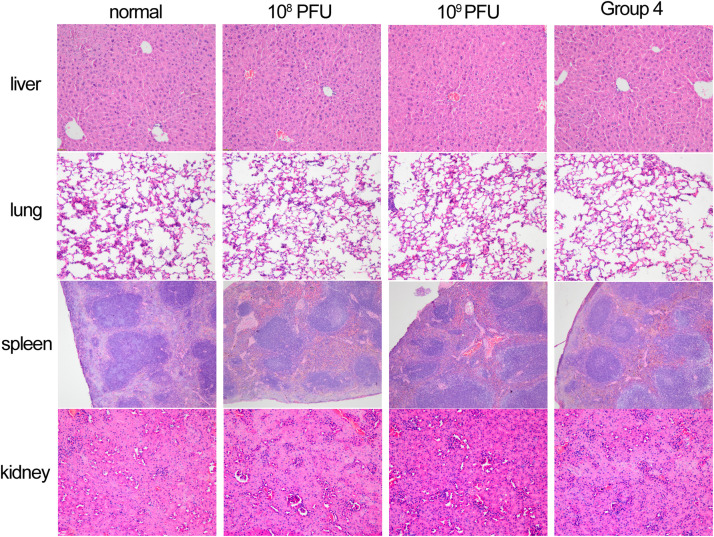
Histopathology of spleen, lung, kidney and liver tissues from normal mouse, mice used to detect the toxicity of different doses of kpssk3 (10^8^ PFU/mouse and 10^9^ PFU/mouse), and phage-rescued mouse.

To evaluate cell viability after treatment with different concentrations of kpssk3, a CCK-8 assay was carried out. As shown in Figure 3, compared to PBS treatment, treatment with 10^8^ PFU/ml or a higher concentration (10^9^ PFU/ml) of kpssk3 had no significant suppressive effect on HeLa cells. For histological examination, the isolated tissue samples from the spleen, lungs, kidneys and liver were stained with hematoxylin and eosin and analyzed under a microscope to determine any pathological changes in the different organs of the mice. As seen in Figure 4, compared to those in the control mice, these 4 organs in the mice treated with different doses of kpssk3 by intraperitoneal injection showed no notable histological changes. In addition, all mice treated with kpssk3 remained alive and healthy during the entire experimental period, without showing any signs of toxicity or any changes in body weight.

### Distribution of kpssk3 in the Blood of Healthy Mice

**FIGURE 5:**
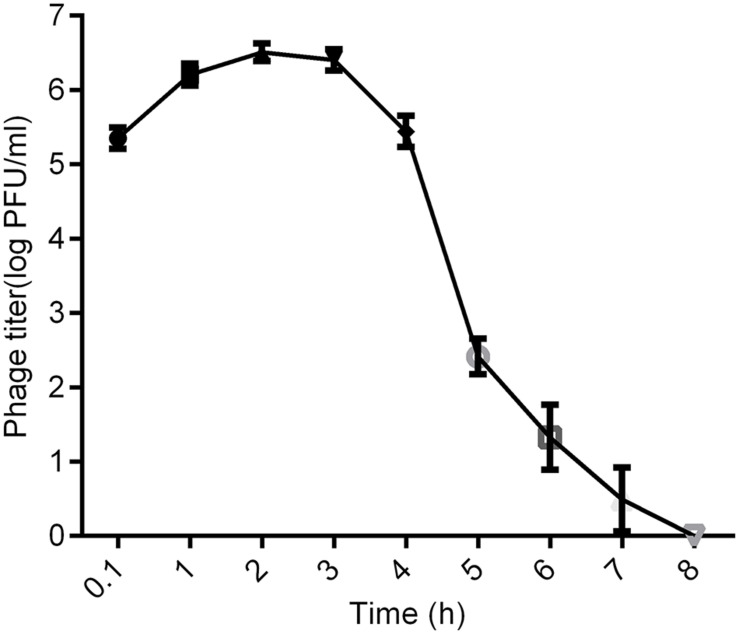
Distribution of kpssk3 in the blood of healthy mice at different time points after intraperitoneal injection with 1 ml of 10^8^ PFU/ml kpssk3.

**FIGURE 6:**
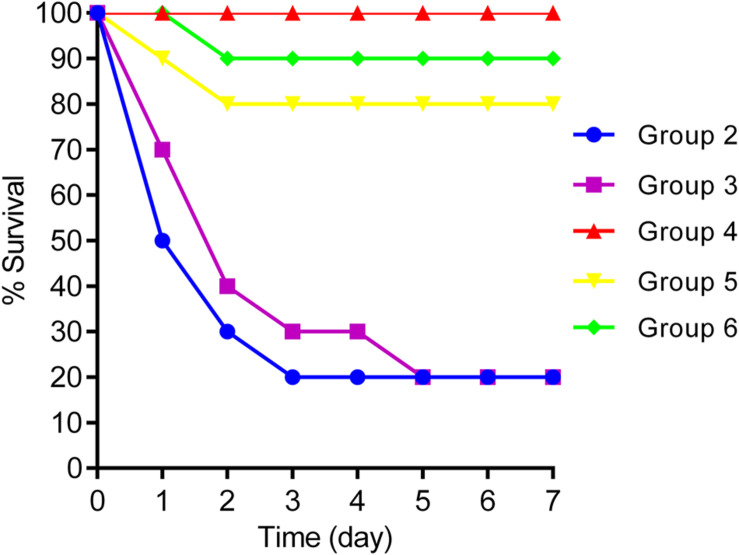
Survival rates for groups 2, 3, 4, 5, and 6.

As shown in Figures 5, 6 min after intraperitoneal injection, kpssk3 was rapidly diffused into the systemic circulation and reached a titer of 10^5^ PFU/ml. During the first 4 h, the blood phage level remained high (approximately 10^6^ PFU/ml) but was approximately two orders of magnitude lower than the injected concentration. Over the subsequent 4 h, the number of active phages in the blood rapidly decreased, completely disappearing by the 8th hour.

### Therapeutic Efficacy of kpssk3 Against Bacteremia in Mice

Intraperitoneal injection of 1 × 10^7^ CFU/mouse of NY03 caused a 100% mortality rate within 1.5 days; therefore, the mouse model of bacteremia was established by infection with 0.1 ml of a log-phase (OD600 = 0.6) bacterial inoculum containing 2 × 10^7^ CFU of NY03. In group 1, intraperitoneal administration of 0.1 ml of NY03 bacterial suspension (2 × 10^7^ CFU/mouse) caused 100% mortality within 8 h. In group 2, the survival rate at the end of the first day was 50% (5/10) and decreased further to 20% (2/10) by the third day, with 2 mice surviving after PMB treatment. In group 3, the survival rate of the mice on day 7 was 20% (2/10); however, treatment with CAZ/AVI plus ATM raised the survival rate of the mice in group 6–90% (9/10). By contrast, 100% of mice infected i.p. with 2 × 10^7^ CFU of NY03 and treated with 10^7^ PFU kpssk3 survived and remained healthy during this entire period. Treatment with 10^6^ PFU kpssk3 protected 80% of mice against bacteremia. No death was observed 7 days after administration of normal saline to mice in group 7. Figure 6, generated with GraphPad Prism (version 6.0), shows the survival rates for groups 2, 3, 4, 5, and 6. By the end of the experiment, no bacteria or phages were found in the blood or organs of the 9 sacrificed mice in group 4. As shown in Figure 4 “Group 4,” histopathological examination of the spleen, lung, kidney and liver removed from the phage-rescued mouse revealed no notable histological changes (Figure 4). And the phage-resistant strain, named NY03r, was obtained by cross-streak method after incubation for about 24 h. NY03r had a same antibiogram with the parent strain (NY03), however, the viscous filament formed by NY03r (approximately 1.5 cm) was much shorter than the one formed by NY03 (Figure 2).

### Alpha Diversity and Beta Diversity

**FIGURE 7:**
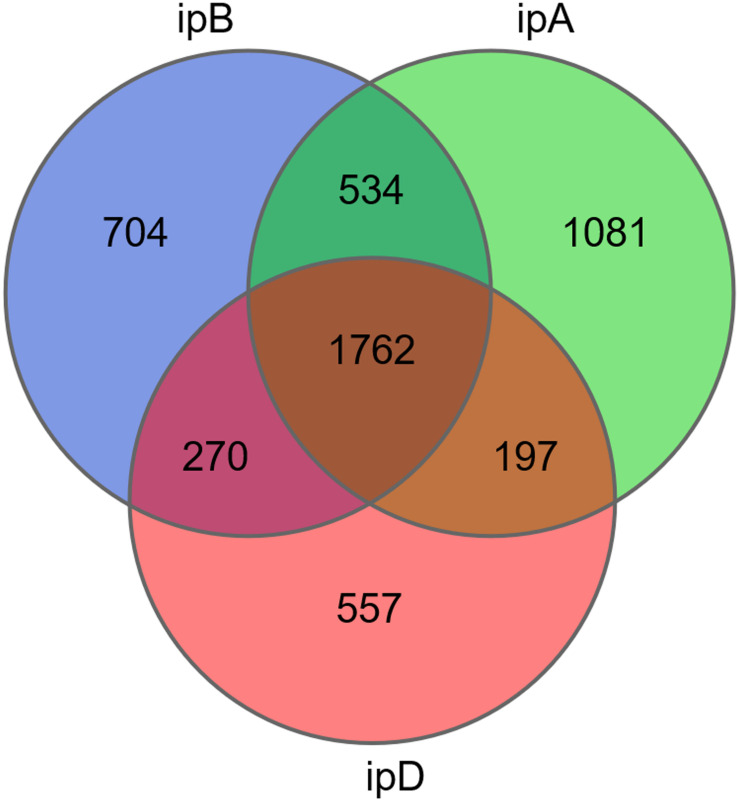
Venn diagram of OTUs in the 3 groups. The different colored circles represent different groups.

The total number of OTUs was 3,270 in the ipB group, 3,574 in the ipA group, and 2,786 in the ipD group, and 1,762 OTUs were shared by the 3 groups (Figure 7). Regarding alpha diversity analysis, the Chao 1, ACE, Shannon, and Simpson indexes of each group are listed in Table 2 and shown with box plots (Supplementary Figure 1). The Chao1 and ACE indexes, which reflect the species richness of samples, were the highest in the ipB group and lowest in the ipD group, and the differences between the 2 groups were statistically significant (Chao1 *P* < 0.05; ACE *P* < 0.05) (Table 3). The Chao1 and ACE indexes in the ipA group were lower than those in the ipB group, but the differences were not significant (*P* > 0.05). The Simpson and Shannon indexes, which reflect the species diversity of samples, did not differ among the 3 groups (*P* > 0.05) (Table 3). Regarding beta diversity analysis, PCoA based on the weighted UniFrac and Bray-Curtis distances revealed that there was no clear separation of the gut microbiome among the ipB, ipA and ipD groups (Supplementary Figure 2). The Adonis test based on the weighted UniFrac or Bray-Curtis distances did not show a significant difference in the gut microbiome between the groups (Table 3). Additionally, we analyzed the microbial composition of each group at different classification levels, including phylum, class, order, family and genus, and selected the top 10 abundant species in each level to generate species relative abundance bar plots (Supplementary Figure 3). The Wilcoxon rank sum test and Kruskal-Wallis test showed no statistically significant differences in the microbial community structure among the 3 groups (Supplementary Table 1).

**TABLE 2:** The mean values of different alpha diversity indices (Chao1, ACE, Shannon, Simpson) in the three groups.

	ipB	ipA	ipD
Chao1	3668.1131	3301.8437	3192.2723
ACE	3648.6512	3264.6104	3206.5178
Shannon	7.0432	7.6565	7.2710
Simpson	0.9670	0.9776	0.9745

**TABLE 3:** Alpha and beta diversity statistical analysis.

Group	α diversity analysis (*p*-value)				β diversity analysis (*p*-value)	
	Chao1	ACE	Shannon	Simpson	Bray−Curtis	Weighted UniFrac
ipB-ipA	0.11	0.14	0.53	0.58	0.54	0.44
ipA-ipD	0.52	0.91	0.97	0.91	0.41	0.33
ipB-ipD	0.01	0.01	0.63	0.68	0.69	0.97
ipB-ipA-ipD	0.04	0.07	0.78	0.82	0.61	0.59

**Alpha diversity indices comparison by Wilcoxon rank sum test or Kruskal-Wallis test; beta diversity indices comparison by Adonis test based on the weighted UniFrac or Bray-Curtis distances.**

## Discussion

CRKP, which is often associated with the lack of available effective antibiotics, increased mortality and exorbitant medical expenses, is an extremely dangerous clinical pathogen. The emergence of and continuing increase in hvKP, which is generally associated with community-acquired invasive infections and the hypermucoviscosity phenotype, constitutes another global threat. Clearly, the combination of hypervirulence and resistance in *K. pneumoniae* clinical isolates may worsen the situation even further (Lai et al., 2019). PT provides us with a promising alternative strategy and new hope in combating hard-to-treat infections. The present study evaluated the therapeutic effects and safety of kpssk3 PT as a treatment for CR-HMKP infection in a mouse model.

The clinical isolate NY03 exhibited the hypermucoviscous phenotype, which is generally considered the hallmark of hypervirulence (Chen et al., 2020). However, large knowledge gaps regarding the relationship between the expression of the capsule, hypermucoviscosity and hypervirulence still exist (Walker et al., 2019). Additionally, there is no consensus definition of hvKP, despite the numerous studies over the past few decades (Cubero et al., 2019). Genomic background and clinical features should be considered when estimating the virulence of a *K. pneumoniae* strain with a positive string test (Catalan-Najera et al., 2017). The tested virulence genes (*rmpA*/*rmpA2*, *magA*, *iucA*, and *iroA*) were not detected in NY03. The mouse lethality assay showed that the LD_100_ of NY03 was 10^7^ CFU, a high inoculation dose compared with that of hvKP strains. Additionally, no invasive infection, such as PLA, lung abscess, endophthalmitis, meningitis or necrotizing fasciitis, was found in the patient who developed sepsis caused by NY03. Therefore, NY03 was considered a low virulent CR-HMKP strain.

To investigate phage kpssk3 as a therapeutic choice, we sought to obtain basic pharmacokinetic data about its distribution in and clearance from the blood of healthy mice. Phages can be delivered via many different administration routes, among which intraperitoneal injection has been proven to be a highly efficient method to introduce phages into the circulatory system (Dabrowska et al., 2005). In this study, active phages were detected in blood 6 min after intraperitoneal administration of kpssk3. The phage titer in the blood peaked within the first hour and remained relatively high for 4 h, which made kpssk3 very applicable for treatment of systemic infections. However, the maximum phage concentration in the blood was approximately 100-fold lower than the injected phage concentration, and no phage was detected in the blood only 8 h after intraperitoneal delivery. Phage kpssk3 was expected to be diluted in blood and cleared by the mononuclear phagocyte system (MPS) after entering the systemic circulation (Dabrowska, 2019). The short life of phages (e.g., kpssk3) in blood has historically been considered an important limitation for PT (Barr, 2017). To change the fate of phages *in vivo*, some experimental attempts (e.g., serial passage technique) have been made to obtain long-circulating phage mutants with an enhanced capability to escape capture by the MPS (Singla et al., 2016), which was favorable in improving the therapeutic potential of PT. However, whether these phage mutants persisting in the circulation can cause adverse effects is unclear. Considering this possibility, short-circulating phages can better prevent the potential side effects of PT.

Notably, the peak blood kpssk3 concentration, approximately 10^6^ PFU/ml, made achieving the inundation threshold (the minimum phage concentration that can reduce the number of sensitive bacteria), which was equal to 10^7^ PFU/ml or higher according to different calculations, difficult (Abedon, 2017). Thus, the phage (kpssk3) must first undergo self-replication, and the titer must then exceed the inundation threshold, based on which the PT in a passive mode can occur and decrease the bacterial population (Cairns et al., 2009). To achieve the optimal therapeutic effect, kpssk3 should be administered at a sufficiently high dose and applied more directly to infections, rather than relying on the concentration of kpssk3 to reach the “killing titer” by replication *in vivo*. In addition, much more research is required to better understand the pharmacological properties of kpssk3, such as its immunogenicity, its distribution in various organs, its ability to cross the blood-brain barrier and placental barrier, and its vertical transmission characteristics.

Regarding the effects of kpssk3, more effective protection of mice from lethal bacteremia was observed in the kpssk3 treatment group than in the PMB or TGC treatment groups, and both kpssk3 and NY03 were fully cleared 7 days later. In addition, the CAZ/AVI plus ATM treatment protocol was used for 7 days, and 90% of mice systemically infected with CR-HMKP survived. On the other hand, the antibiotic treatment protocols used in this study may need to be optimized for the selection of antibiotics, dosage, combination therapy and the prevention of side effects. Regarding the safety of kpssk3 PT, kpssk3 had no cytotoxic effect on mammalian cells and a lower impact on the gut microbiota. Many studies have demonstrated that the gut microbiota plays significant roles in human health (Ahlawat et al., 2020). Introduction of phages into the blood is followed by rapid extravasation of the phages from the bloodstream into internal organs and other tissues, including the gastrointestinal tract (Barr, 2017). Therefore, we are concerned about the short- and long-term impacts of kpssk3 treatment on the gut microbiota, despite its high specificity. Based on 16S rDNA analysis, only the Chao1 and ACE indexes were significantly decreased 10 days after kpssk3 PT, possibly because of the emergence of slight gut microbiota dysbiosis induced by PT. In addition, the possibility that NY03 impacted the gut microbiota cannot be ruled out. Whether this change is restored after 10 days requires further study. Obviously, we need to remain vigilant about the potential side effects and long-term effects of PT.

In summary, treatment with phage kpssk3 increased the survival rate of mice with CR-HMKP-induced bacteremia to 100%, and no serious side effects or signs of toxicity were observed in the *in vivo* test. There were also no significant changes in gut microbiome of mice during the treatment period. Based on these results, kpssk3 PT could be considered an effective alternative for CR-HMKP bacteremia due to its considerable therapeutic effect and lack of side effects.

## Data Availability Statement

The data presented in the study are deposited in the NCBI SRA repository, accession number PRJNA723119 (https://www.ncbi.nlm.nih.gov/Traces/study1/?acc=PRJNA723119).

## Ethics Statement

The animal study was reviewed and approved by the Ethics Committee of Army Medical University.

## Author Contributions

YLG and YXZ conceived and designed the experiments. YLS, YP, and YC performed the experiments. YJC and ZQY analyzed the data. CZ, XQL and JC contributed reagents and materials. YLS wrote the paper. All authors contributed toward revising the paper and agreed to be accountable for all aspects of the work.

## Conflict of Interest

The authors declare that the research was conducted in the absence of any commercial or financial relationships that could be construed as a potential conflict of interest.

## Footnotes

1 https://github.com/OpenGene/fastp

## Supplementary Material

Supplementary Figure 1 Box plot of the alpha diversity indexes (Chao1, ACE, Shannon, and Simpson).

Supplementary Figure 2 Principal coordinates analysis (PCoA) plot based on Bray-Curtis **(A)** and weighted UniFrac distances **(B)**. Each point represents the microbial community structure of an individual fecal sample.

Supplementary Figure 3 Relative abundance of species at different classification levels (phylum, class, order, family, genus). The abscissa shows grouping information, and the ordinate shows the relative abundance.
