# Supplementary material for: Safety and Efficacy of a Phage, kpssk3, in an in vivo Model of Carbapenem-Resistant Hypermucoviscous Klebsiella pneumoniae Bacteremia
Source: Front Microbiol. 2021 May 20;12:613356. doi: 10.3389/fmicb.2021.613356 (PMC8175031; doi:10.3389/fmicb.2021.613356)
Supplement: Supplementary file 1 [file Table_1.DOCX]

**Supplementary** Table 1. The relative abundance of dominant bacterium at different classification levels in 3 groups and the statistical analysis.

| **Phylum** | **Group** | | |  | **P value** | | | |
| --- | --- | --- | --- | --- | --- | --- | --- | --- |
|  | **ipB(%)** | **ipA(%)** | **ipD(%)** |  | **ipB-ipA-ipD** | **ipB-ipA** | **ipB-ipD** | **ipA-ipD** |
| Bacteroidetes | 69.052 | 59.411 | 67.931 |  | 0.47 | 0.25 | 0.52 | 0.63 |
| Firmicutes | 17.951 | 17.543 | 18.145 |  | 0.78 | 0.63 | 0.58 | 0.79 |
| Proteobacteria | 5.815 | 8.218 | 6.602 |  | 0.24 | 0.14 | 0.19 | 0.85 |
| Actinobacteria | 2.406 | 6.641 | 2.118 |  | 0.32 | 0.16 | 0.53 | 0.39 |
| Planctomycetes | 0.833 | 1.454 | 0.894 |  | 0.33 | 0.12 | 0.48 | 0.68 |
| Acidobacteria | 0.769 | 1.290 | 0.704 |  | 0.50 | 0.25 | 0.68 | 0.57 |
| Gemmatimonadetes | 0.546 | 1.298 | 0.465 |  | 0.39 | 0.16 | 0.97 | 0.39 |
| Chloroflexi | 0.591 | 0.910 | 0.602 |  | 0.51 | 0.28 | 0.48 | 0.74 |
| Cyanobacteria | 0.309 | 1.056 | 0.461 |  | 0.31 | 0.14 | 0.53 | 0.43 |
| Saccharibacteria | 0.253 | 0.482 | 0.401 |  | 0.83 | 0.63 | 0.63 | 0.97 |

| **Class** | **Group** | | |  | **P value** | | | |
| --- | --- | --- | --- | --- | --- | --- | --- | --- |
|  | **ipB(%)** | **ipA(%)** | **ipD(%)** |  | **ipB-ipA-ipD** | **ipB-ipA** | **ipB-ipD** | **ipA-ipD** |
| Bacteroidia | 68.311 | 58.372 | 67.264 |  | 0.47 | 0.25 | 0.53 | 0.63 |
| Clostridia | 15.770 | 16.059 | 16.916 |  | 0.61 | 0.58 | 0.35 | 0.73 |
| Actinobacteria | 1.509 | 5.559 | 1.356 |  | 0.27 | 0.14 | 0.63 | 0.27 |
| Gammaproteobacteria | 2.284 | 3.024 | 2.227 |  | 0.65 | 0.35 | 0.74 | 0.73 |
| Betaproteobacteria | 1.465 | 1.530 | 1.833 |  | 0.26 | 0.14 | 0.21 | 0.91 |
| Bacilli | 1.747 | 1.122 | 0.987 |  | 0.85 | 0.74 | 0.79 | 0.63 |
| Alphaproteobacteria | 0.781 | 1.931 | 0.778 |  | 0.41 | 0.22 | 0.74 | 0.39 |
| Deltaproteobacteria | 0.905 | 1.159 | 0.943 |  | 0.62 | 0.58 | 0.31 | 0.97 |
| Gemmatimonadetes | 0.472 | 1.204 | 0.424 |  | 0.33 | 0.12 | 1 | 0.39 |
| Planctomycetacia | 0.507 | 0.751 | 0.529 |  | 0.43 | 0.19 | 0.53 | 0.73 |

| **Order** | **Group** | | |  | **P value** | | | |
| --- | --- | --- | --- | --- | --- | --- | --- | --- |
|  | **ipB(%)** | **ipA(%)** | **ipD(%)** |  | **ipB-ipA-ipD** | **ipB-ipA** | **ipB-ipD** | **ipA-ipD** |
| Bacteroidales | 68.306 | 58.371 | 67.261 |  | 0.47 | 0.24 | 0.52 | 0.63 |
| Clostridiales | 15.766 | 16.045 | 16.913 |  | 0.61 | 0.57 | 0.35 | 0.73 |
| Pseudonocardiales | 0.412 | 2.365 | 0.418 |  | 0.27 | 0.16 | 0.35 | 0.39 |
| Xanthomonadales | 0.796 | 1.181 | 0.809 |  | 0.62 | 0.34 | 0.73 | 0.63 |
| Lactobacillales | 1.372 | 0.794 | 0.528 |  | 0.59 | 0.85 | 0.73 | 0.24 |
| Burkholderiales | 0.691 | 0.926 | 0.876 |  | 0.38 | 0.24 | 0.27 | 0.85 |
| Pseudomonadales | 0.633 | 0.780 | 0.772 |  | 0.48 | 0.39 | 0.27 | 0.97 |
| Gemmatimonadales | 0.472 | 1.204 | 0.424 |  | 0.33 | 0.12 | 1 | 0.39 |
| Planctomycetales | 0.507 | 0.751 | 0.529 |  | 0.43 | 0.19 | 0.52 | 0.73 |
| Enterobacteriales | 0.584 | 0.602 | 0.413 |  | 0.42 | 0.31 | 0.85 | 0.25 |

| **Family** | **Group** | | |  | **P value** | | | |
| --- | --- | --- | --- | --- | --- | --- | --- | --- |
|  | **ipB(%)** | **ipA(%)** | **ipD(%)** |  | **ipB-ipA-ipD** | **ipB-ipA** | **ipB-ipD** | **ipA-ipD** |
| Bacteroidales_S24-7_group | 49.452 | 39.097 | 49.528 |  | 0.44 | 0.27 | 1 | 0.31 |
| Lachnospiraceae | 8.552 | 8.738 | 10.262 |  | 0.24 | 0.35 | 0.12 | 0.43 |
| Prevotellaceae | 7.327 | 9.378 | 9.911 |  | 0.36 | 0.21 | 0.68 | 0.31 |
| Ruminococcaceae | 5.828 | 5.439 | 5.396 |  | 0.77 | 0.52 | 0.63 | 1 |
| Rikenellaceae | 5.694 | 5.355 | 3.904 |  | 0.58 | 0.52 | 0.73 | 0.35 |
| Bacteroidaceae | 3.075 | 2.289 | 2.132 |  | 0.51 | 0.52 | 0.31 | 0.57 |
| Porphyromonadaceae | 2.355 | 1.713 | 1.438 |  | 0.62 | 0.57 | 0.57 | 0.35 |
| Pseudonocardiaceae | 0.412 | 2.365 | 0.418 |  | 0.27 | 0.16 | 0.35 | 0.39 |
| Lactobacillaceae | 1.271 | 0.689 | 0.468 |  | 0.76 | 0.79 | 0.85 | 0.43 |
| Gemmatimonadaceae | 0.472 | 1.204 | 0.424 |  | 0.33 | 0.12 | 1 | 0.39 |

| **Genus** | **Group** | | |  | **P value** | | | |
| --- | --- | --- | --- | --- | --- | --- | --- | --- |
|  | **ipB(%)** | **ipA(%)** | **ipD(%)** |  | **ipB-ipA-ipD** | **ipB-ipA** | **ipB-ipD** | **ipA-ipD** |
| Alloprevotella | 3.452 | 3.818 | 4.326 |  | 0.71 | 0.63 | 0.91 | 0.43 |
| Prevotellaceae_UCG-001 | 2.571 | 3.307 | 4.037 |  | 0.45 | 0.52 | 0.19 | 0.85 |
| Lachnospiraceae_NK4A136_group | 2.717 | 3.128 | 3.425 |  | 0.51 | 0.43 | 0.27 | 1 |
| Rikenellaceae_RC9_gut_group | 3.197 | 3.242 | 2.511 |  | 0.71 | 0.57 | 0.79 | 0.48 |
| Bacteroides | 3.075 | 2.289 | 2.132 |  | 0.51 | 0.52 | 0.31 | 0.57 |
| Alistipes | 2.131 | 1.752 | 1.066 |  | 0.56 | 0.63 | 0.73 | 0.27 |
| Odoribacter | 2.123 | 1.394 | 1.257 |  | 0.80 | 0.68 | 0.52 | 0.97 |
| Ruminococcaceae_UCG-014 | 0.905 | 0.945 | 0.809 |  | 0.17 | 0.052 | 0.79 | 0.27 |
| Lactobacillus | 1.271 | 0.689 | 0.468 |  | 0.76 | 0.79 | 0.85 | 0.43 |
| Ruminiclostridium | 1.231 | 0.506 | 0.674 |  | 0.74 | 0.73 | 0.97 | 0.39 |
