# Supplementary figures and images for: Safety and Efficacy of a Phage, kpssk3, in an in vivo Model of Carbapenem-Resistant Hypermucoviscous Klebsiella pneumoniae Bacteremia
Source: Front Microbiol. 2021 May 20;12:613356. doi: 10.3389/fmicb.2021.613356 (PMC8175031; doi:10.3389/fmicb.2021.613356)

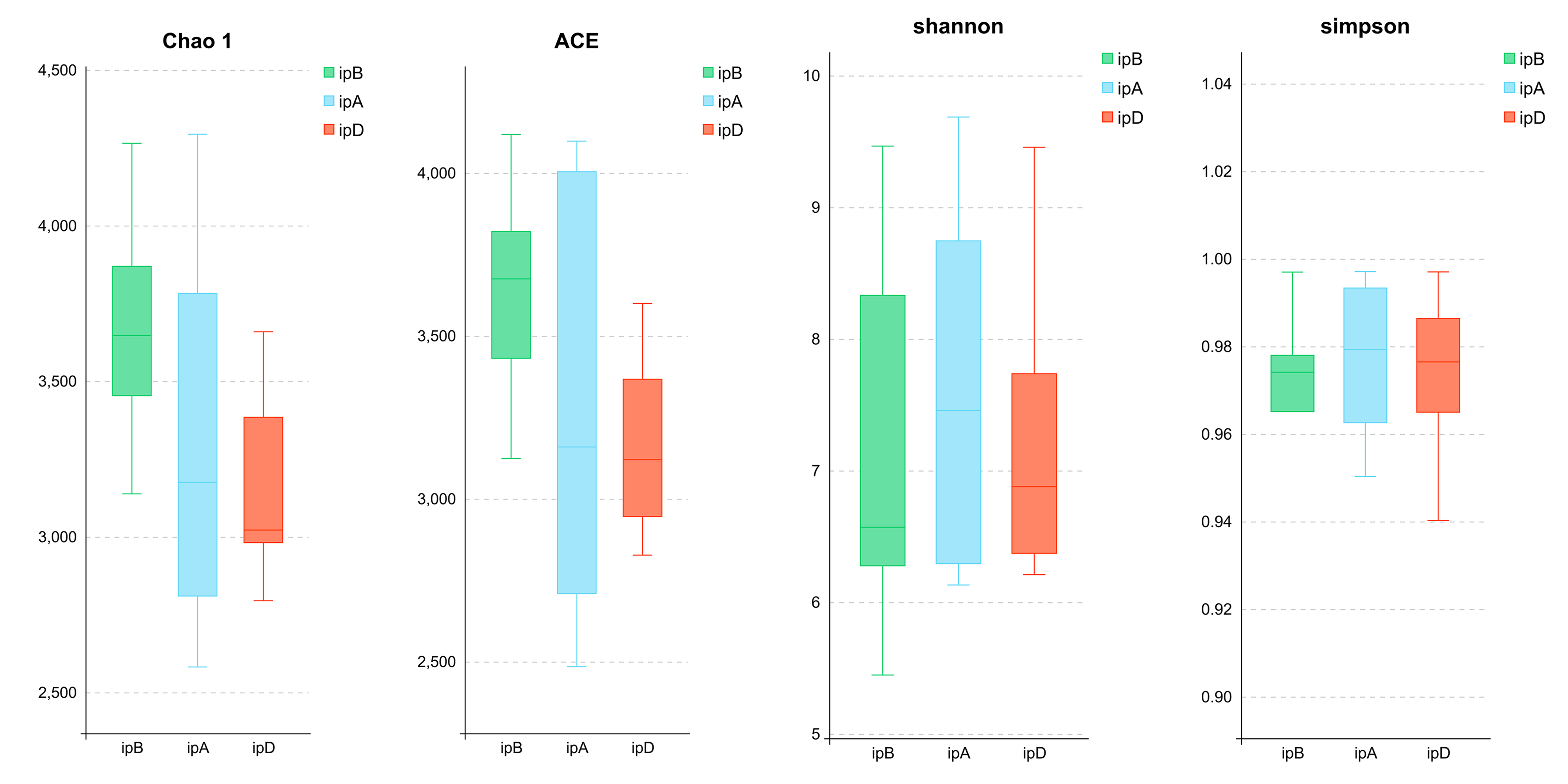

Supplement: Supplementary Figure 1 — Box plot of the alpha diversity indexes (Chao1, ACE, Shannon, and Simpson). [file Image_1.TIF]

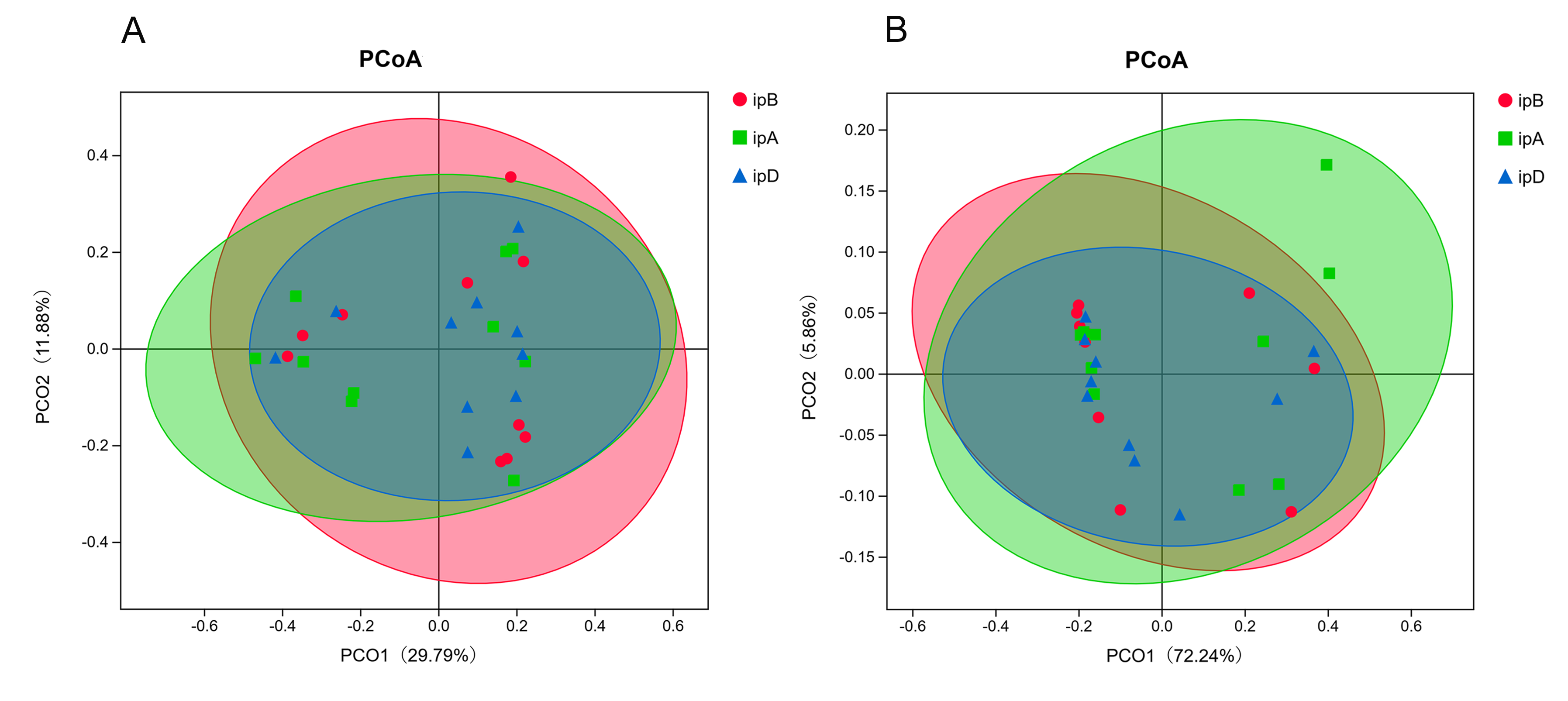

Supplement: Supplementary Figure 2 — Principal coordinates analysis (PCoA) plot based on Bray-Curtis (A) and weighted UniFrac distances (B). Each point represents the microbial community structure of an individual fecal sample. [file Image_2.TIF]

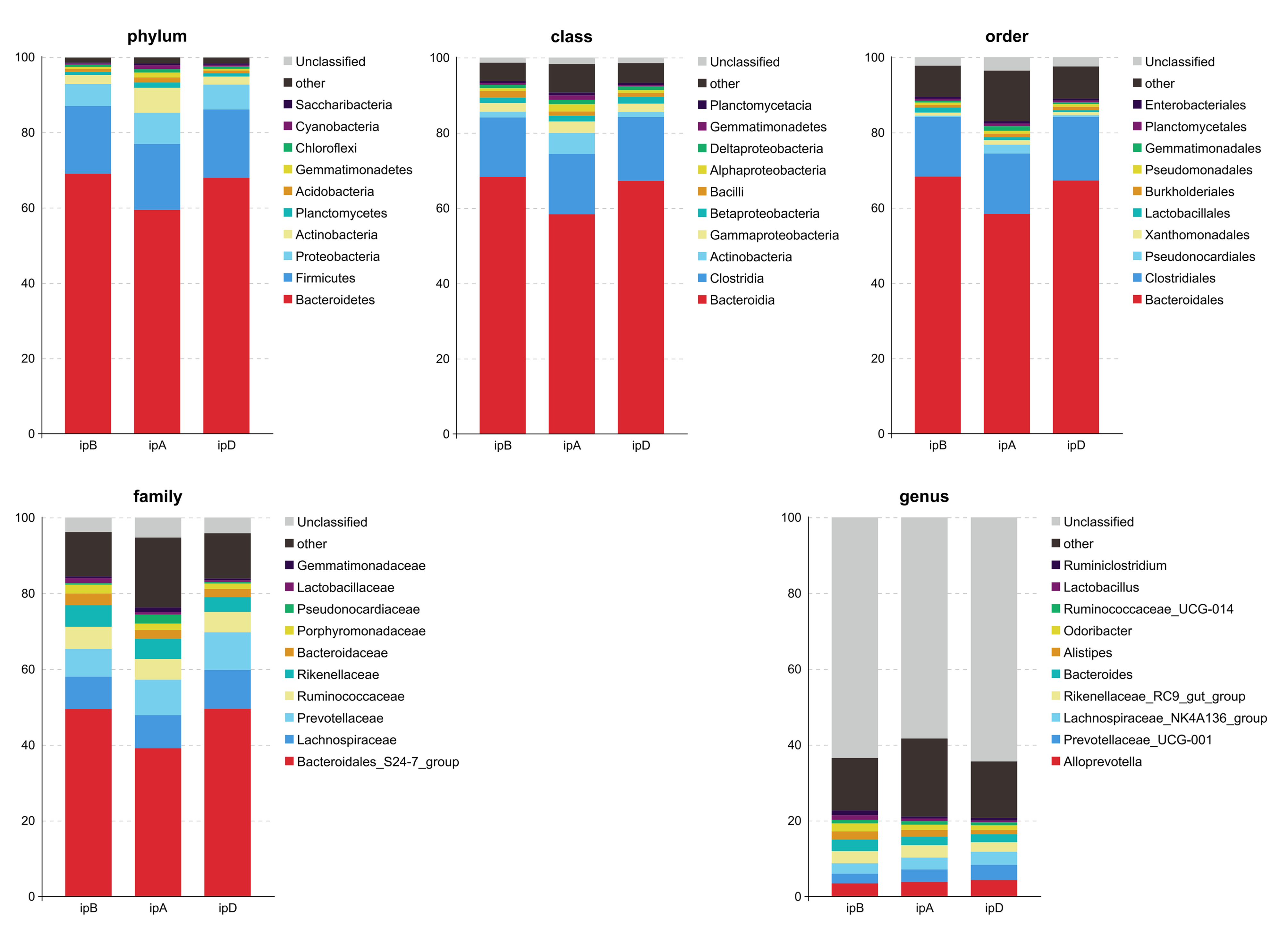

Supplement: Supplementary Figure 3 — Relative abundance of species at different classification levels (phylum, class, order, family, genus). The abscissa shows grouping information, and the ordinate shows the relative abundance. [file Image_3.TIF]
